# Linking the sender to the receiver: vocal adjustments by bats to maintain signal detection in noise

**DOI:** 10.1038/srep18556

**Published:** 2015-12-22

**Authors:** Jinhong Luo, Holger R. Goerlitz, Henrik Brumm, Lutz Wiegrebe

**Affiliations:** 1 Max Planck Institute for Ornithology, Acoustic and Functional Ecology Group, Eberhard-Gwinner-Straße, 82319 Seewiesen, Germany; 2 Division of Neurobiology, Department Biology II, Ludwig-Maximilians-Universität München, Großhaderner Straße 2, 82152 Planegg-Martinsried, Germany; 3 Max Planck Institute for Ornithology, Communication and Social Behaviour Group, Eberhard-Gwinner-Straße, 82319 Seewiesen, Germany

## Abstract

Short-term adjustments of signal characteristics allow animals to maintain reliable communication in noise. Noise-dependent vocal plasticity often involves simultaneous changes in multiple parameters. Here, we quantified for the first time the relative contributions of signal amplitude, duration, and redundancy for improving signal detectability in noise. To this end, we used a combination of behavioural experiments on pale spear-nosed bats (*Phyllostomus discolor*) and signal detection models. In response to increasing noise levels, all bats raised the amplitude of their echolocation calls by 1.8–7.9 dB (the Lombard effect). Bats also increased signal duration by 13%–85%, corresponding to an increase in detectability of 1.0–5.3 dB. Finally, in some noise conditions, bats increased signal redundancy by producing more call groups. Assuming optimal cognitive integration, this could result in a further detectability improvement by up to 4 dB. Our data show that while the main improvement in signal detectability was due to the Lombard effect, increasing signal duration and redundancy can also contribute markedly to improving signal detectability. Overall, our findings demonstrate that the observed adjustments of signal parameters in noise are matched to how these parameters are processed in the receiver’s sensory system, thereby facilitating signal transmission in fluctuating environments.

Many animals rely on acoustic information for mate attraction, social integration, conflict resolution, predator-prey interaction, or orientation^1^. However, the transmission of acoustic signals is severely constrained by environmental noise, which in turn imposes strong selection pressure on sound production and perceptual mechanisms^2^. To mitigate signal masking, animals often adjust their vocalizations to the characteristics of background noise^2^. A particularly well studied form of noise-dependent vocal plasticity is the Lombard effect. The Lombard effect is the increase in vocal amplitude of a subject in response to an increase in the background noise level^3^,^4^,^5^, which is a basic mechanism for maintaining communication in noise in birds and mammals, including humans^4^,^5^,^6^. The Lombard effect is often accompanied by changes in other signal parameters such as the spectral energy distribution or the duration of vocalizations^4^,^5^.

The relationship between the Lombard effect and other noise-induced vocal modifications has attracted continual research effort since the discovery of the Lombard effect over a century ago^3^,^4^,^5^,^6^,^7^. Nevertheless, the contribution of changes in different signal parameters to reduce noise interference has only been quantified in a few studies^8^,^9^. Lu and Cooke (2009) found that flattening of spectral tilt in noise contributed greatly to improve speech intelligibility while an increase in fundamental frequency did not have an influence^8^. In a modelling study, Nemeth and Brumm (2010) demonstrated that the Lombard effect is more effective in mitigating masking from traffic noise in urban bird songs than noise-related changes in song frequency^9^.

In contrast, most studies on animal vocal production in noise that investigated multiple acoustic parameters have not examined how the interaction of these parameters affects the receiver’s capacity to detect the signal. A particular relevant combination of vocal parameters in vertebrates is the amplitude, duration and redundancy (or signal repetition) of the signal, because they are integrated by a fundamental auditory process in the receiver: temporal summation^10^,^11^. It is the combined effect of these parameters, instead of each parameter in isolation, which determines the detectability of a signal by the receiver. In other words, due to temporal summation, the detectability of brief sound signals increases with amplitude, duration, and the number of signal repetitions within short time intervals^11^,^12^,^13^,^14^. Thus, to maintain a given signal detectability, an animal could either increase signal amplitude, signal duration, or signal redundancy, or any combination thereof. In birds and mammals, the integration window for temporal summation extends up to a few hundred milliseconds^11^,^15^,^16^, which makes temporal summation a crucial feature for signal detection of many types of natural vocalizations^17^,^18^,^19^. However, it remains unclear whether these parameters are equally efficient in reducing noise interference and whether animals make equal use of them for maintaining signal detectability in noise.

We studied the relative contributions of signal amplitude, signal duration and signal redundancy for the improvement of signal detectability in a highly vocal bat species (*Phyllostomus discolor*) which produces short echolocation calls ranging from 0.3 to 2.5 ms in duration^20^. Echolocation is an active sensing mechanism by which animals (e.g. bats, toothed whales) probe their environment by producing high-frequency, usually ultrasonic, vocalizations and listen to the returning echoes to represent their surroundings^21^. Bat echolocation calls are highly flexible in signal structure and often tuned precisely to the task at hand^22^,^23^. Moreover, bats also adjust the structure of their echolocation calls to interfering sounds such as conspecific calls^24^ and artificial noise^25^,^26^.

We recorded echolocation calls and used perceptual models of sound detection to quantify the relative contribution of signal amplitude, duration and redundancy for improving signal detectability in noise. In a mimicked roosting context, we recorded echolocation calls of three pairs of bats exposed to background noise at three different frequency ranges (noise types) and sound pressure levels (Fig. 1). The noise types included both non-overlapping noise (10–35 kHz) and overlapping noise (40–90 kHz and 10–90 kHz) in relation to the bats’ typical echolocation call frequencies (36–100 kHz). Each noise type was broadcast at 28, 40, and 52 dB SPL, respectively. We thus used nine noise conditions, with each noise condition being defined as a combination of noise level and noise type, plus an additional silence control. In a second step, the observed noise-induced changes in call characteristics were used to calculate changes in signal detection probabilities based upon a signal detection model that combines the physical properties of sound signals and the receivers’ perception.

## Results

### Effects of noise type and noise level on signal parameters

Figure 2a shows exemplary calls from the silence condition (left) and the 52 dB SPL 10–90 kHz noise condition (right) respectively, exemplifying the typical pattern of calls being longer and higher in amplitude in the noise condition than in the silence condition. For the two noise types that overlapped in frequency with the echolocation calls, bats gradually increased the amplitude of their echolocation calls with increasing noise level (Fig. 2b). The average increase in signal root mean square (RMS) level reached up to 4.6 dB (in the 52 dB SPL 10–90 kHz noise condition). Similarly, bats produced longer calls with increasing noise level in both overlapping noise types, with an average increase of 0.37 ms (i.e. 1.5 fold) and 0.4 ms (i.e. 1.6 fold) at the 52 dB noise level (Fig. 2c). For the noise type that did not overlap in frequency with the echolocation calls, the changes in signal amplitude and signal duration were much smaller, with an average increase of 1.7 dB for signal amplitude and of 0.04 ms for signal duration.

We found that the noise-induced changes in signal amplitude and signal duration were highly variable across individuals (Fig. 3a,b; Supplementary Figure S1). For example, in the 52 dB SPL 10–90 kHz noise condition, the average increase in signal amplitude ranged from 1.8 to 7.9 dB in different individuals (Fig. 3a). Similarly, the average increase in signal duration ranged from 1.1 (Bat 6) to 1.8 (Bat 4) folds (Fig. 3b). In contrast, for the non-overlapping noise type there was no systematic increase in signal amplitude or signal duration with increasing noise level (Supplementary Figure S2).

### Effects of vocal adjustments on signal detectability

The probability of successfully detecting a short sound increases with both signal amplitude and duration^11^. Thus, it is the combined effect of signal amplitude and signal duration, namely signal detectability per call that determines the detection threshold. Using the Leaky Integration, Event Formation, Temporal Summation (LIEFTS) model^27^, we quantified signal detectability for each echolocation call (for details see Methods). We found that signal detectability per call was qualitatively similar to the performance of both signal amplitude and signal duration (Figs 2c and 3c; Supplementary Figures S1c, S2c). Specifically, for both overlapping noise types, the signal detectability per call increased gradually with increasing noise level (Fig. 2c). For the non-overlapping noise type, signal detectability only increased in the highest noise level condition by 1.9 dB, although much less than in the overlapping noise types. This effect might be accounted for by an upward spread of masking into higher frequency bands at high masker intensities^10^.

To quantify the contribution of signal duration to signal detectability, we subtracted for each individual and for each noise condition the contribution of the Lombard effect, i.e. the contribution of signal amplitude, from the signal detectability per call and described the remaining difference as a function of signal duration, using linear least squares regression. We found that a doubling of signal duration increased signal detectability on average by 6 dB (95% confidence interval: 5.5–6.5 dB; Fig. 4a, Pearson correlation, *R*^2^ = 0.96, *P* < 0.001), as predicted by the model for signal durations between 0.5 and 2 ms (5.7–6.9 dB, τ = 1.5 ms; Fig. 4b). Thus, the maximum noise-dependent increase in signal duration in the 10–90 kHz noise type (1.8 fold, Fig. 3b, Bat 1 and Bat 4) resulted in an increase in signal detectability of 5.1 dB. Across all bats, the maximum increase in signal duration was 1.6 fold, which resulted in an increase in signal detectability of 3.3 dB, or 3.7 dB (95% confidence interval: 3.4–4.1 dB) based on the regression function above (Fig. 4a).

Bats organized their calls into groups (Fig. 5a). Calls within a group were separated by shorter call intervals (CI) compared to the CIs between groups. The CI histogram had a strong peak at CIs <50 ms, containing 61% of all calls, and a long right-skewed tail (Fig. 5b). Using 50 ms as criterion to separate call groups (see Methods for details) we found that bats on average produced 3 calls per group across all noise conditions (Fig. 5c). Overall, about 75% of the calls originated from call groups; the remainder were single calls. Since calls within a call group are separated by less than 50 ms, they might be integrated by temporal summation, and thus can increase signal detectability by increasing signal redundancy. In addition to analysing signal detectability for single calls (see above), we therefore also quantified signal detectability for call groups. Specifically, the relative detectability of single calls was multiplied by the ratio of the average call number of a call group from a noise condition to the silence control. Overall, signal redundancy within call groups contributed only 1–2% to signal detectability for both overlapping noise types, whereas the increase in signal duration yielded 41–46%, and the increase in signal amplitude (the Lombard effect) 52–57% (Fig. 6). We also found that bats produced more call groups in the 10–90 kHz overlapping noise type than in the silence control (Fig. 5d). In particular, the number of call groups per minute was on average 4 times greater in the 52 dB SPL 10–90 kHz condition than in the silence control. Assuming optimal cognitive integration^13^, this increase of signal redundancy due to more call groups could result in a further detectability improvement by up to 4 dB relative to the silence condition.

## Discussion

Psychophysicists have long been aware of the importance of the relationship between amplitude and duration for signal detection, a phenomenon referred to as temporal summation^10^,^16^. In recent years, a growing number of studies have applied this concept to explain why animals increase signal duration in noise^19^,^26^,^28^,^29^,^30^,^31^. Likewise, an increase in signal redundancy in noise has also been interpreted as a response to counteract noise interference^32^,^33^,^34^. Here, we quantified for the first time the relative contribution of signal amplitude, duration, and redundancy for improving signal detectability. Our results show that both the Lombard effect and the adjustment of signal duration are important in improving signal detectability in noisy environments.

In contrast to the Lombard effect, which has been reported in all tested species of birds and mammals including humans^4^,^5^, changes in signal duration in noise are much more varied between species and cover all three possibilities of an increase^19^, a decrease^35^, and no change^36^. Likewise, while all bats in our experiment exhibited the Lombard effect, changes in signal duration were more variable across individuals. Quantifying the relative contribution of signal amplitude and signal duration for our bats, revealed that increasing signal duration improved signal detectability substantially, but generally weaker than the Lombard effect. The maximum contribution by signal duration was 5.1 dB, compared to the maximum contribution of 7.9 dB by the Lombard effect. Averaged across all animals, increases in signal duration resulted in an average increase in signal detectability of 3.3 dB, compared to 4.6 dB by the Lombard effect.

The magnitude of the Lombard effect ranges from a few to more than a dozen decibels in different species^4^,^6^. In comparison, many studies in different species reported a less than twofold increase in signal duration^19^,^26^,^29^,^30^,^37^. Similarly, we found that signal duration increased maximally by a factor of 1.8 in the tested bats. However, so far it has been unclear how much detectability animals can gain by doubling the duration of their vocalizations. Our current model suggests that the benefit for signal detectability from increasing signal duration depends strongly on the absolute signal duration. Specifically, animals using short vocalizations of only a few milliseconds gain much more detectability per doubling of signal duration than animals using longer vocalizations (about 6 dB vs. 2 dB; Fig. 4b). In psychophysical experiments on temporal integration, free-tailed bats (*Tadarida brasiliensis*) improved the detectability of their calls by about 6 dB in when signal duration was increased from 2 to 4 ms^38^. This is in quantitative agreement with our current model. Our model also correctly predicts the observation in free-tailed bats that further doublings of signal duration to 8 ms and longer durations does not result in equally strong improvements^38^. This might explain why three other bat species with call durations of only a few milliseconds increased their signal duration in noise^26^,^39^,^40^, whereas horseshoe bats with call durations of 40–50 ms did not^25^,^41^.

A few animal species have been reported to respond to interfering noise by increasing their signal redundancy^32^,^33^,^34^,^42^. Greater signal redundancy can improve signal detectability through two distinct processes depending on the type of background noise. First, repeating the same signal will increase the probability of one of these repetitions to occur within a relative silent period of fluctuating background noise^34^. In turn, receivers are able to capture brief acoustic glimpses of a signal during relative silent periods of background noise^43^,^44^. Second, repeating the same signal allows receivers to perform ‘multiple looks’, i.e. an increase of performance based on peripherally independent detection events^13^,^14^,^45^,^46^,^47^. The bats in our experiment did not produce more calls per call group in the presence of noise, but increased the number of call groups in the 10–90 kHz overlapping noise type.

It is unclear, however, to which extent bats may benefit from signal redundancy for improving signal detectability. Since, to our knowledge, no data is available on the effect of signal redundancy on signal detectability in bats, we limited our calculations to calls within a call group based on the available psychophysical evidence in humans^13^,^14^,^47^,^48^ and cats^45^. A second reason for this limit was that there is growing evidence that bats integrate information from multiple calls within a call group during natural echolocation tasks^49^,^50^. On the other hand, our finding that bats increased the number of call groups with increasing noise level in the 10–90 kHz overlapping noise type raises the possibility that bats might also integrate information across call groups. The bats produced about 4 times more call groups in the 52 dB SPL 10–90 kHz noise condition than in silence. Assuming that bats are capable of optimally integrating information across call groups within an entire six-minute data acquisition period, the increase in signal detectability due to call-group redundancy could amount to 4 dB^13^. However, this type of cognitive integration of the redundancy provided by multiple call groups is physiologically different from the hardwired temporal summation adopted for the within-group analyses, and its physiological foundations are unknown.

The present study has focused on how amplitude and temporal properties of acoustic signals improve signal detectability in noise, with an estimated average increase in signal detectability of 8 dB in the 52 dB SPL 10–90 kHz noise condition compared to the silence control (Fig. 2d). However, it is important to note that this increase in detectability is not sufficient to fully compensate for the increase of masking by the background noise, which increased by more than 24 dB from silence to the highest noise level condition. This means that echo detection in all noise conditions will be worse than in the silence condition and that the observed increase in detectability will only partially compensate for the impairment of echo detection.

Besides amplitude and temporal properties, animals may also change the spectral properties of their vocalizations^5^,^51^,^52^, and it has been suggested that spectral plasticity is used to reduce signal masking by noise^7^,^53^,^54^. In this study, bats exhibited a gradual decrease in bandwidth (measured at −10 dB below the peak frequency level) with increasing noise level, with a maximum reduction of 3.4 kHz in the 10–90 kHz overlapping noise type (Supplementary Figure S3), which was caused by a stronger decrease of the maximum frequency than the minimum frequency. Noise-related reduction in bandwidth has also been observed in bird vocalizations^55^ and, like increasing signal amplitude or signal duration, it improves signal detectability by concentrating more signal energy into given auditory filters^56^. However, to our best knowledge, no published signal detection model can so far integrate temporal and spectral features of vocalizations, which is why we excluded spectral changes from our model. Compared to increased amplitude, however, the effect of spectral changes is probably rather small, as suggested by the five times larger effect of the Lombard effect on bird communication distance in traffic noise, compared to spectral changes^9^.

In summary, this study demonstrated how three typical sound parameters, signal amplitude, signal duration, and signal redundancy, are integrated by temporal summation to determine overall signal detectability. Particularly for animals that emit very short signals of only a few milliseconds, such as many species of echolocating bats, not only increasing call amplitude, but also increasing signal duration can substantially contribute to improving signal detectability in noise. We emphasize that due to temporal summation the combined effect of all three signal parameters determines signal detectability in noise. We therefore suggest that signal detectability, not a single call parameter, is the principal target for vocal adjustments in noise.

## Methods

### Animals and setup

**Figure 1:**
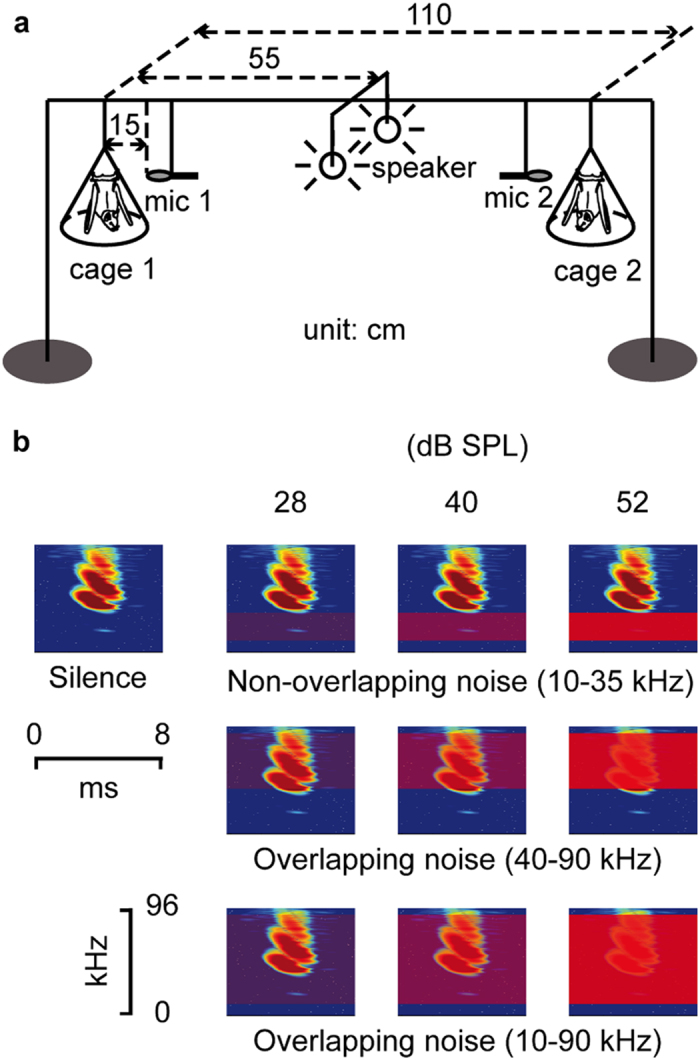
Experimental setup and artificial noise. (**a**) Experimental setup (not to scale). The vocalizations of two bats, one in each cage, were recorded by microphones while presenting uncorrelated band-pass filtered white noise through two omnidirectional loudspeakers. (**b**) Illustrations of the spectrogram of artificial noise in relation to a typical echolocation call of *Phyllostomus discolor*. Noise was presented at three different sound pressure levels (28, 40 and 52 dB SPL re. 20 μPa RMS) and frequency bands (10–35, 40–90 and 10–90 kHz), which were either non-overlapping or overlapping with the bats’ typical range of call frequencies (36–100 kHz). During the silence control, the noise was digitally switched off.

**Figure 2:**
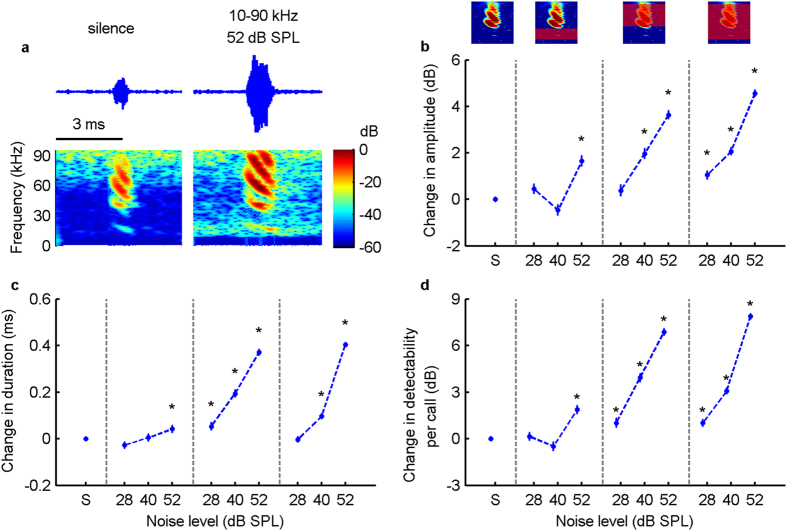
Adjustments of signal parameters and signal detectability of bat echolocation calls in noise. (**a**) One exemplary echolocation call from the silence condition (left) and one from the 52 dB SPL 10–90 kHz noise condition (right). Note that the microphone was directed away from the noise-presenting speakers, thus not capturing the noise spectrum as received by the bat, particularly for higher frequencies. The noise above 60 kHz is caused by intrinsic electronic high-frequency noise of the recording system and is not audible to the bat. (**b**,**c**) Change in call amplitude and call duration, pooled for all six bats, and presented as differences (marginal mean and 95% confidence interval) in relation to the silence control (S).The marginal means in the silence control were 68.3 dB SPL (re. 20 μPa RMS) for signal amplitude and 0.73 ms for signal duration. (**d**) Signal detectability per call is the combined effect of signal amplitude and signal duration. The number of analysed calls for each condition from left (S) to right (52 dB SPL 10–90 kHz condition) was 30,779, 6,145, 5,042, 5,052, 4,833, 5,438, 7,935, 8,492, 10,382 and 15,439. Asterisks (*) above data points indicate a statistical difference from the silence control that is of potential biological relevance (see Methods for details).

**Figure 3:**
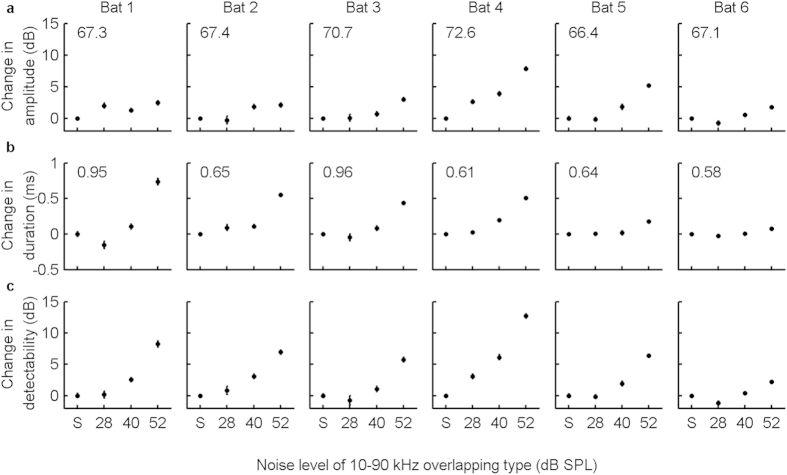
Adjustments of signal parameters and signal detectability of bat echolocation calls in the 10–90 kHz overlapping noise type per individual. Data are presented as differences (mean and 95% confidence interval) in relation to the silence control (S). Numbers in the top left corner of each panel of (**a**,**b**) are the means of signal amplitude (dB SPL re. 20 μPa RMS) and signal duration (ms) for each individual in the silence control. The number of analysed calls for each individual from left (Bat 1) to right (Bat 6) was 6,438, 6,480, 12,105, 22,708, 7,627 and 9,734.

We tested six adult *Phyllostomus discolor* (Wagner 1843), three males and three females. The bats were housed in a holding room, with regular food supply and *ad libitum* access to water. We conducted the experiment in an echo- and sound-attenuated acoustic chamber. During the experiment, bats were held individually in pyramidal mesh cages (30 cm high, 30 × 30 cm at the base, and 10 × 10 cm at the top, Bat World Sanctuary, Weatherford, USA) while recording their vocalizations with microphones (CO 100K, Sanken, Saitama, Japan) at 15 cm distance (Fig. 1a). Uncorrelated noise was broadcast from two omnidirectional loudspeakers (Elac 4PI PLUS.2, Elac Electroacoustic, Kiel, Germany) placed 5 cm apart in the centre between the bats. This experiment was conducted under the principles of laboratory animal care and the regulations of the German Law on Animal Protection. As the experiment is neither invasive nor stressful, it does not require explicit approval according to the regulations. The license to keep and breed *P. discolor* was issued by the responsible agency (Regierung von Oberbayern, Germany).

### Experimental paradigm

The six individuals were tested in three pairs (one male-male, one female-female and one male-female pair), to which they were assigned for the full duration of the experiment of 18 days. On each experimental day, we collected data from all 3 pairs in random order for a period of 40 minutes per pair. The 40-minute test session consisted of a four-minute habituation phase at the beginning, followed by three six-minute noise treatments and three six-minute silence treatments, in alternating order. The type and order of noise treatments per day and pair were assigned via blockwise randomization. Per day and pair, each noise type and each noise level was presented once, and all possible nine noise treatments (i.e., combination of noise type and level) were presented once within three days, resulting in six repetitions of all noise treatments after 18 experimental days.

The purpose of testing two bats simultaneously was to study the Lombard effect of both echolocation calls and social calls. However, the tested bats produced very few social calls in this experiment and thus we focused on echolocation calls here.

### Recording and playback

Sound recording and noise playback were synchronized through an audio interface (Ultralite-mk3 Hybrid, MOTU, Cambridge, USA) which was controlled by SoundMexPro software (HörTech, Oldenburg, Germany) in MATLAB (Version 7.5, The MathWorks Inc., Natick, MA, USA). We monitored all vocalizations produced during the entire test sessions at a sampling rate of 192 kHz. Whenever any of the two microphones received a signal with peak amplitude above the trigger threshold (70 dB SPL relative to 20 μPa), recordings of 1.9 s before and 0.1 s after the trigger event were saved to hard disc. If two consecutive recordings overlapped, the overlapping part was analyzed only once.

We used three types of uncorrelated band-pass filtered white noise (20th order infinite impulse response (IIR) filter), each of which was broadcast from both speakers at three levels: 28, 40, and 52 dB SPL re. 20 μPa RMS (Fig. 1b). The effective noise level received by the bats was about 3 dB higher for each noise condition due to the summation of two uncorrelated noise sources. For the silence control condition, the noise level was digitally set to − 20 dB SPL. The first noise type covered frequencies between 10–35 kHz, and did not overlap with the frequency range of the bats’ echolocation calls, in contrast to the other two noise types, 40–90 kHz and 10–90 kHz noise (Fig. 1b), which both overlapped spectrally with the bat calls. The band-pass filter for the non-overlapping noise type resulted in a sharp amplitude decrease of 23 dB at 40 kHz. For higher frequency components, the amplitude decrease is much larger. Thus, there is essentially no leaking of sound energy for the non-overlapping noise type into echolocation call frequencies. The entire frequency range of all three noise types is audible to the bats^57^. However, the perceived loudness of the three noise types for the bats probably differed due to the uneven frequency sensitivity of the bats’ hearing. Since the exact frequency sensitivity depends on the measurement method^57^, we did not compensate for potential differences in perceived loudness.

We ensured a flat frequency response of the noise playback system by filtering the noise with each speaker’s compensatory impulse response (511-order finite impulse response (FIR) filter with cut-off frequencies of 7 and 90 kHz). Initial speaker measurements were conducted with an 1/8 inch measurement microphone (Type 4138, Brüel & Kjær, Nærum, Denmark; protective grid removed) oriented perpendicular to each speaker at a distance of 55 cm (i.e., at the position of the bat). For each noise treatment and for each speaker, uncorrelated white noise was generated, filtered by the respective noise type, convolved with the compensatory impulse response of each speaker, and played to the bats continuously for 6 minutes per noise treatment. The convolution with the compensatory impulse responses resulted in a flat frequency spectrum (±1 dB) of the playback system in the pass-band.

### Sound analysis

We performed all sound analyses in MATLAB with custom-made programs based on Goerlitz *et al.* 2008^58^. First, we accounted for the frequency response of the microphones by filtering the recorded call with each microphone’s compensatory impulse response (32^th^ order FIR filter) and then high-pass filtered all recordings at 35 kHz (5^th^ order IIR filter). All echolocation calls with a peak amplitude >=70 dB SPL (i.e., the trigger threshold of the recording) were automatically identified by the software, followed by a manual graphical check of the waveform, the spectrogram and the power spectrum to ensure call identification quality. In total, 99,537 echolocation calls from six bats were identified and analyzed, with a median of 1,158 calls per individual and noise condition (range: 150–11,891). For each echolocation call, we extracted its duration (measured at a threshold of −10 dB below the peak of the envelope obtained from a Hilbert transform), RMS amplitude over the call duration, and signal detectability. Bandwidth was the difference between the maximum and minimum frequency at −10 dB below the peak frequency in the power spectrum. The number of calls per group and the number of call groups per minute were calculated for each six minute treatment. Call groups were defined based on the call interval. Call groups of bats are often characterized by relatively stable and short call intervals (intra-group intervals), while different call groups are separated by longer call intervals (inter-group interval)^49^,^50^(Fig. 5a). The distribution of call intervals in our study peaked at 33 ms, with a median of 39 ms (Fig. 5b), and a long right-skewed tail. We defined a call group as consisting of all calls separated by less than 50 ms. Note that we only used call groups with at least two calls and excluded single calls from the redundancy analysis.

### Signal detectability

Recent studies showed that across a variety of vertebrate species, the signal detection threshold at both neuronal and perceptual levels can be well represented by the Leaky Integration, Event Formation, Temporal Summation (LIEFTS) model^15^,^18^,^27^. The LIEFTS model posits that sound detection is a physiological process, which sums individual detection events over the signal duration, with the individual probability of detection being proportional to the third power of the time-varying output of the Leaky Integration in the auditory system^27^. As a result, the output of this physiological process, the summed detection events, reflects how likely a receiver may successfully detect a sound signal. We refer to the quantity of the summed detection events for a sound signal as signal detectability. Signal detectability thus represents a quantity at the perceptual level, which combines the physical properties of sound signals and the receiver’s perception.

**Figure 4:**
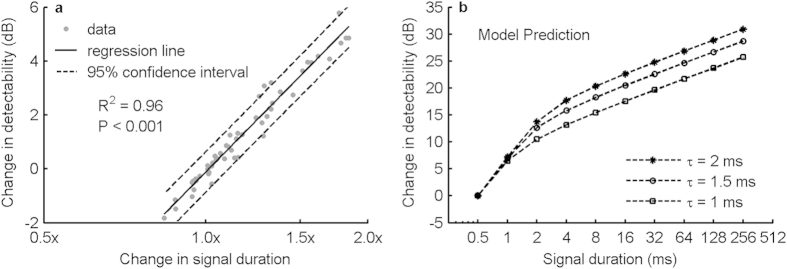
Increased signal duration results in increased signal detectability. (**a**) Changes in signal detectability due to changes in signal duration were calculated by subtracting the Lombard effect (Fig. 3a) from the overall change in signal detectability per call (Fig. 3c). Each data point represents the vocal performance of one individual in one noise condition, with a total of 54 data points from 6 bats and 9 noise conditions excluding silence control. Values on the x-axis are the changes in signal duration between noise treatment (T) and silence control (T_0_) and are represented as ratios (T/T_0_). (**b**) Effect of signal duration on temporal summation, predicted by the LIEFTS model for three time constants (*τ*). Note that increasing the length of short signals of a few milliseconds is more effective in improving signal detectability than of longer signals.

We adapted the LIEFTS model to compute changes in signal detectability of short echolocation calls. The absolute signal detectability is


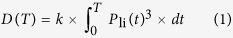


where *D*(*T*) is the signal detectability of a call with duration *T*, *k* is the species-specific parameter of the Event Formation step, and *P*_li_(*t*) is the leaky integrated pressure envelope of the time-varying signal (*P*(*t*)) obtained from Hilbert transform


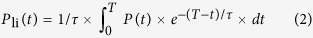


where *τ* is a short time constant (about 1–2 ms), which can be thought of to represent the passive properties of the membrane of the inner hair cells^27^. Thus, *P*_li_(*t*) is the low-pass filtered stimulus envelope. Applying the LIEFTS model to the detection threshold data of humans confirmed *τ* to be 1.56 ms and 1.8 ms for two data sets^15^,^18^. Thus, in this study, *τ* was set to 1.5 ms. The effect of *τ* between 1 ms and 2 ms on model predictions was systematically evaluated and presented in Fig. 4b.

Event Formation depends on the species-specific parameter *k*. Determining *k* requires perceptual data which is often unknown for a given species. However, in many cases not the absolute value of signal detectability is of interest, but its relative change caused by different physical sound properties in different situations (e.g., between noisy and silent conditions). When calculating the relative change Δ*D*, this species-specific parameter *k* is eliminated (see equation (4) below), which allows applying this model to a wide range of species. Since *k* does not affect Δ*D*, it can be set to any arbitrary value. In this study, we calculated arbitrary absolute sound detectability of each recorded call with *k* set to 1. For each individual, we then calculated the median arbitrary absolute signal detectability in the silence control 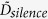 as a baseline for comparison with the calls emitted in the noise conditions.

To compare signal detectability of each call emitted in a noise condition to the silence control, we expressed Δ*D* in decibels (dB) per call, based on the equation to calculate the sound pressure level difference Δ*p* in decibel between two sound pressures *P*_*1*_ and *P*_*2*_


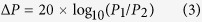


Signal detectability estimated by equation (1) is based on the third power of the pressure envelope. To substitute *P* in equation (3) with signal detectability, the 3^rd^ root of signal detectability has to be used^15^,^18^. Thus, for each call from the noise conditions, we obtained Δ*D* by comparing the 3^rd^ root of its detectability *D*(*T*) to the 3^rd^ root of the median signal detectability in the silence condition 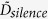 by


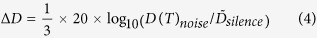


Temporal summation operates not only one single sound signals, but also across multiple signals separated by short time intervals^13^,^45^,^46^,^47^,^48^. The contribution of signal redundancy for improving signal detectability can also be quantified by a modified version of the LIEFTS model^12^. As a result, in addition to the change in signal detectability computed for single calls by equation (4), we also calculated the change in signal detectability per group of calls by


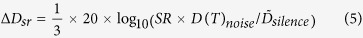


where 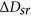 is the magnitude of noise compensation in dB after accounting for signal redundancy (*SR*). Signal redundancy in this particular model is expressed as the ratio of the average number of calls per group for each noise condition to the average number of calls per group in the silence condition. Effectively, equation (5) approximates the change in signal detectability due to changes in the summed total duration of multiple calls between two conditions.

**Figure 5:**
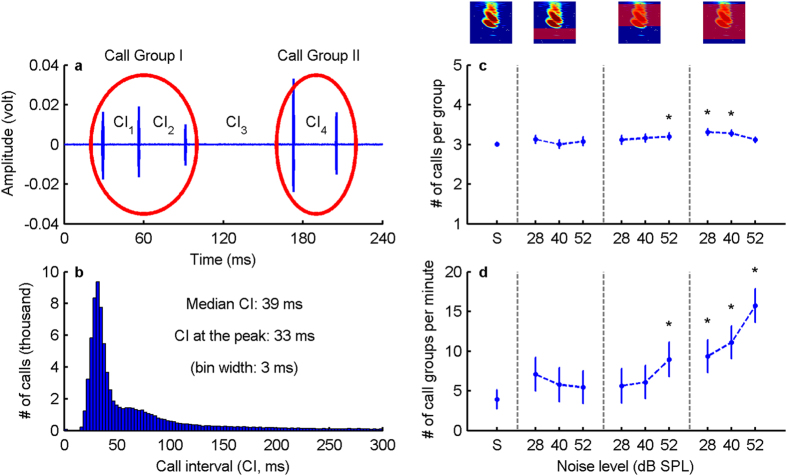
Number of calls per minute and number of call groups per minute in different noise conditions. (**a**) Example recording from a silence condition, with two call groups and their respective call intervals (CI). (**b**) Call intervals pooled across all noise conditions and bats. (**c,d**) Number of calls per group and number of call groups per minute, separated by noise conditions, presented as marginal means and 95% confidence intervals. Asterisks (*) above data points indicate a statistical difference from the silence control (*P*_*adj*_ < 0.01, Bonferroni adjusted *P*-value for multiple comparison).

**Figure 6:**
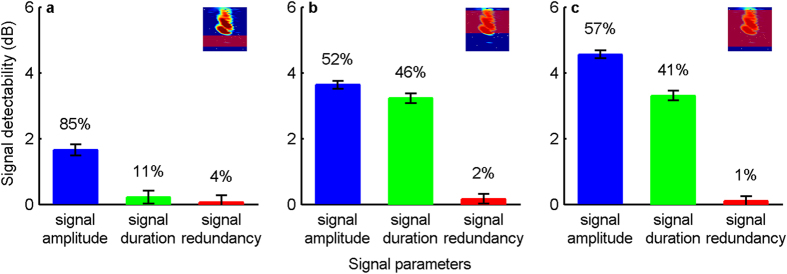
Relative contributions of noise-induced vocal modifications to signal detectability. Changes in signal detectability are shown for the 52 dB SPL (highest amplitude) noise conditions, and presented as marginal mean and 95% confidence interval. Numbers above each bar show the relative contribution of this parameter to signal detectability within a call group.

The bats on average produced three calls per call group and the median inter-call interval is 39 ms (Fig. 5). Thus, the time interval integrated for calls within a call group is about 80 ms. Both the total signal duration and the gaps between calls are well within the thresholds of temporal summation in literature^12^.

### Model evaluation

The LIEFTS model was originally developed to explain the stimulus dependence of first-spike latency of auditory-nerve fibres^27^. Subsequently, it has been successfully applied to fit the psychophysical detection threshold data of both humans and birds^15^,^18^. Although spontaneous firing rate is a critical parameter in explaining the first-spike latency of auditory–nerve fibres of high spontaneous firing rate^27^, this parameter is not required when fitting the detection threshold data^15^,^18^. Here we adapted the LIEFTS model to calculate the changes in signal detectability due to changes in acoustic properties of sound signals, i.e. changes in detection threshold. As explained above, one benefit of this approach is to enable us to skip the step of determining the species-specific parameter *k* in equation (1), which raises the concern whether our approach can fit the psychophysical data used to prove the LIEFTS model. As is shown in Supplementary Figure S4 online, our way of using the LIEFTS model fits the human psychophysical detection threshold data^59^ quite well. Only when the signal duration is longer than about 300 ms, the model prediction shows a systematic overestimation by about 2 dB for the longest signal duration of 1065 ms. As explained by the original authors of the LIEFTS model, this deviation is probably due to lower attention by the tested subjects^15^.

### Amplitude threshold for call selection

One methodological limitation when studying vocal communication in noise is that weak calls buried in background noise are missed. Here, we could not analyze calls with peak amplitudes below 70 dB SPL, raising the question whether excluding relative faint calls might have affected our conclusions. To address this question, we raised the amplitude threshold for call selection from 70 to 80 dB SPL in 1 dB steps and repeated all analyses, which we present in the Supplementary Figure S5 online, showing only small changes in the details and confirming our overall results. Throughout the main paper, all results are based on the 70 dB SPL amplitude threshold for call selection. At the 80 dB SPL amplitude threshold, the minimum call number per individual and condition dropped to a median of 294 (range: 5–5,909), preventing us from increasing the threshold further.

### Statistics

We modelled signal amplitude, signal duration, signal bandwidth, the number of calls per group, the number of call groups per minute, as well as signal detectability (per call, and per group), as a function of *noise condition* and *individual identity* (if data from all bats were included) respectively, using Linear Models run in SPSS 21.0 (IBM Corp., USA). A *noise condition* was defined as a combination of noise level and noise type, resulting in 10 noise conditions including the silence control. All parameters were modelled using *identity* link function. Both *noise condition* and *individual identity* were set as fixed factors, and only the main effects were investigated (i.e. without the interaction effect). The model fits were examined by subsequent analyses of the residuals. All *P*-values for pair-wise comparisons reported in this paper were adjusted with Bonferroni correction and were denoted with *P*_*adj*_. Note, however, that *P*-values should be considered in conjunction with effect size^60^ when interpreting the presented results, because statistical sensitivity increases with sample size and our analyses were based on a large dataset of 99,537 calls. Thus, we further justified whether changes in a signal parameter is of potential biological relevance in terms of perceptual thresholds. Specifically, potential biological relevance refers to the situation where, upon statistical significance, changes in signal amplitude/detectability were greater than 1 dB, or changes in signal duration were larger than 5%. The specific criteria of 1 dB for amplitude/detectability and 5% for duration are close to the minimum psychophysical thresholds of discrimination^10^.

## Additional Information

**How to cite this article**: Luo, J. *et al.* Linking the sender to the receiver: vocal adjustments by bats to maintain signal detection in noise. *Sci. Rep.* **5**, 18556; doi: 10.1038/srep18556 (2015).

## Supplementary Material

Supplementary Information
