# Supplementary material for: Linking the sender to the receiver: vocal adjustments by bats to maintain signal detection in noise
Source: Sci Rep. 2015 Dec 22;5:18556. doi: 10.1038/srep18556 (PMC4686984; doi:10.1038/srep18556)

Supplementary information

Linking the sender to the receiver: vocal adjustments by bats to maintain signal detection in noise

Jinhong Luo, Holger R. Goerlitz, Henrik Brumm, Lutz Wiegrebe

Supplementary Figure S1

Adjustments of signal parameters and signal detectability of bat echolocation calls in the 40-90 kHz overlapping noise type per individual. Data are presented as differences (mean and 95% confidence interval) in relation to the silence control (S). Numbers in the top left corner of each panel of (**a**) and (**b**) are the means of signal amplitude (dB SPL re. 20 μPa RMS) and signal duration (ms) for each individual in the silence control. The number of analysed calls for each individual from left (Bat 1) to right (Bat 6) was 3,857, 5,842, 8,449, 19,530, 2,782, and 8,525.

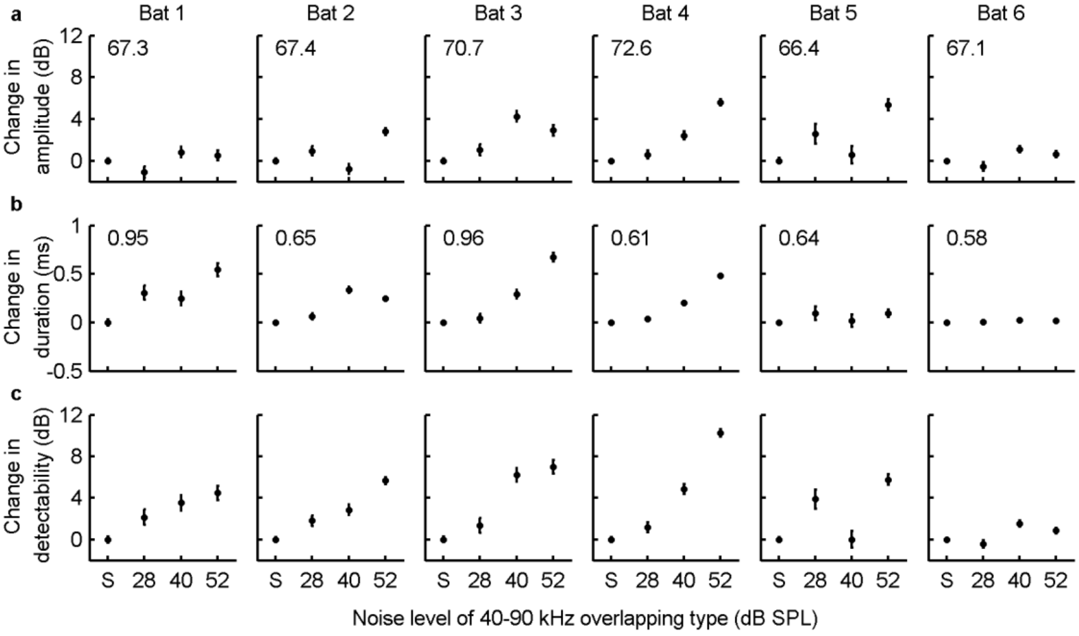

Supplementary Figure S2

Adjustments of signal parameters and signal detectability of bat echolocation calls in the 10-35 kHz non-overlapping noise type per individual. Data are presented as differences (mean and 95% confidence interval) in relation to the silence control (S). Numbers in the top left corner of each panel of (**a**) and (**b**) are the means of signal amplitude (dB SPL re. 20 µPa RMS) and signal duration (ms) for each individual in the silence control. The number of analysed calls for each individual from left (Bat 1) to right (Bat 6) was 3,839, 4,484, 9,781, 18,848, 2,875, and 7,191.

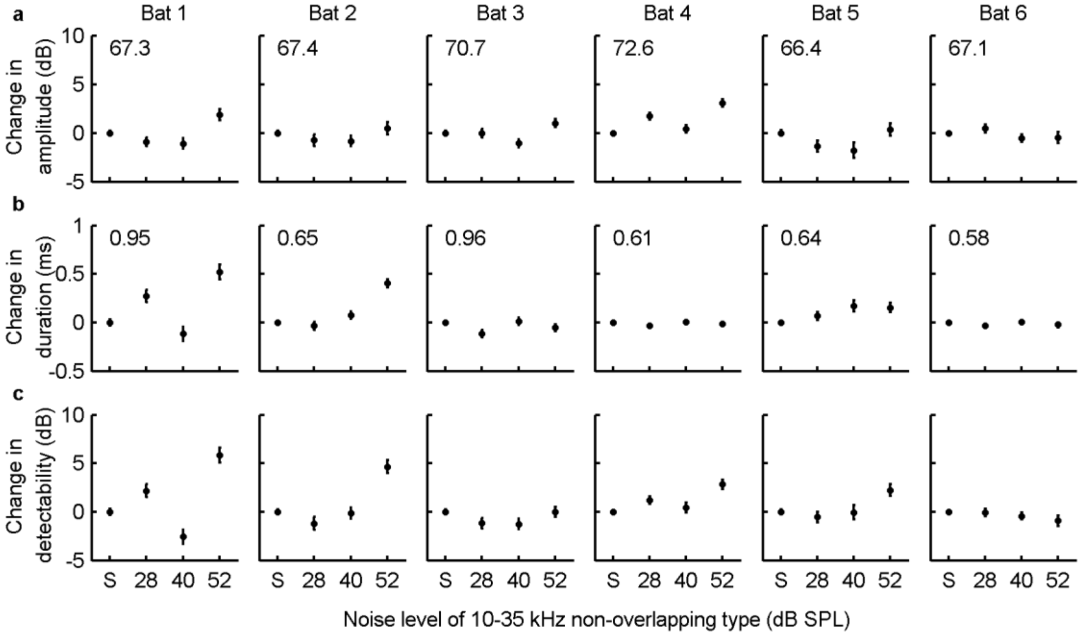

Supplementary Figure S3

Noise-induced changes in -10 dB signal bandwidth of *Phyllostomus discolor* echolocation calls. Data (marginal mean and 95% confidence interval) were pooled for all six bats and presented as changes in relation to the silence control (S). Asterisks (*) above data points indicate a statistical difference from the silence control ($P_{adj} < 0.01$, Bonferroni adjusted P-value for multiple comparison).

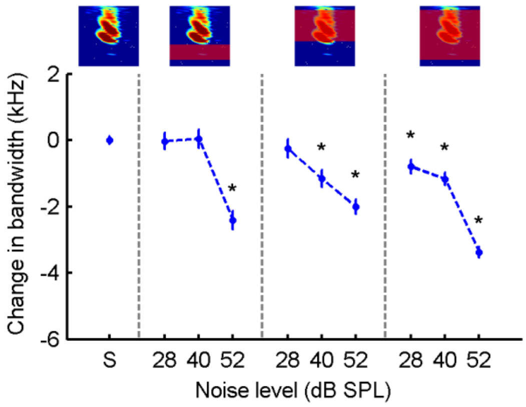

Supplementary Figure S4

LIEFTS model validation with human psychophysical detection threshold data from Heil et al. 2006. The same psychophysical data set has been fitted with the LIEFTS model by Heil and his colleagues in two publications (Heil et al. 2013; Pohl et al. 2013). Here we used the same time constant (τ) as Pohl et al 2013 to predict the relative detection thresholds (blue square), and compared them with measured detection thresholds (red asterisk). It shows that the LIEFTS model predicted the relative detection thresholds quite well for signal durations less than about 300 ms. For the longest signal duration of 1,065 ms, the model overestimated the detection threshold by about 2 dB, which is probably due to the attention effects of the tested subjects as explained by Heil et al. 2013.

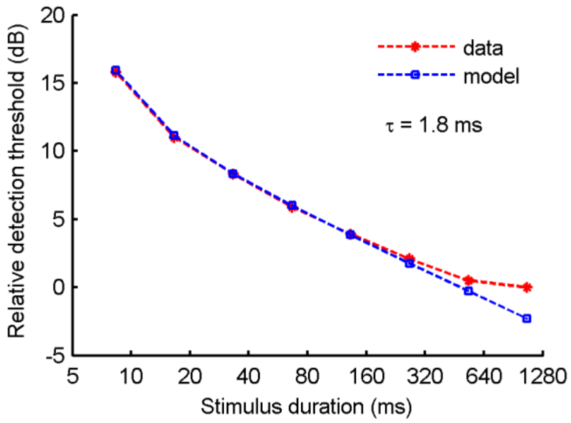

Supplementary Figure S5

Effects of call selection threshold on the magnitude of the Lombard effect and on its contribution to signal detectability. Data were pooled for all six bats. (**a**) The Lombard effect as a function of call selection threshold. (**b**) Relative contribution of signal amplitude (blue), signal duration (green), and signal redundancy (red) to signal detectability in relation to call selection threshold. Numbers above each bar of (**c**) show the relative contribution of the Lombard effect to signal detectability.

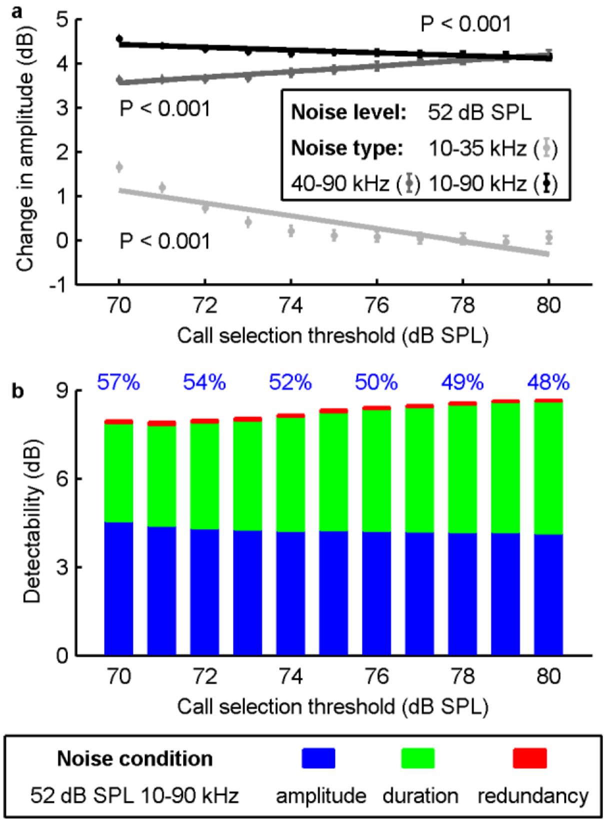

Supplement: Supplementary Information [file srep18556-s1.pdf]
